# Case Report: Clinical and histopathological findings of porcelain gallbladder in a dog

**DOI:** 10.3389/fvets.2025.1570221

**Published:** 2025-05-20

**Authors:** Tae-Young Kim, Ye-In Oh

**Affiliations:** ^1^Jukjeon Animal Medical Center, Dalgubeol-daero, Daegu, Republic of Korea; ^2^Department of Veterinary Internal Medicine, College of Veterinary Medicine and Institute for Veterinary Biomedical Science, Kyungpook National University, Daegu, Republic of Korea

**Keywords:** porcelain gallbladder, calcification, fibrosis, gallbladder mucocele, cholecystitis

## Abstract

Porcelain Gallbladder (PGB) is a condition marked by extensive calcification and thickening of the gallbladder wall. PGB is extremely rare in dogs. The exact mechanism of PGB remains unclear. However, chronic cholecystitis and factors such as biliary hyperplasia, mucin hypersecretion, and cystic duct obstruction can lead to fibrosis and calcification of the gallbladder wall, potentially resulting in PGB. A 13-year-old spayed female Shih Tzu presented with anorexia, lethargy, vomiting, and weight loss. Physical exams showed mild epigastric pain. Blood tests indicated mild alanine aminotransferase (ALT) elevation, significant alkaline phosphatase, and gamma-glutamyl transferase (GGT) increases. Hyperlipidemia, hypercholesterolemia, and elevated canine pancreatic lipase (cPL) were also noted. Diagnostic imaging revealed extensive gallbladder wall calcification and thickening, choleliths, mild bile duct dilation, pancreatitis, and duodenitis. Cholecystectomy was performed, and symptomatic treatment for pancreatitis and duodenitis was administered. Histopathological examination is essential for confirming PGB by identifying calcification of the gallbladder wall. Histopathological examination of the gallbladder revealed severe papillary hyperplasia, mucin hypersecretion, multiple ulcers, diffuse calcification, and fibrotic changes. These findings confirmed the diagnoses of gallbladder mucocele and PGB. Postoperatively, biochemical markers normalized or significantly decreased, with clinical improvement observed. This study presents the rare occurrence of PGB in dogs and emphasizes the importance of appropriate veterinary intervention for improving clinical outcomes.

## 1 Introduction

Porcelain Gallbladder (PGB) is defined as calcification of the gallbladder wall, characterized by extensive calcification and thickening (1, 2). There are various hypotheses on the mechanism that causes PGB; however, its etiology remains unknown (3–11). Some authors maintain that it is an unusual manifestation of chronic cholecystitis (3, 12, 13). Chronic cholecystitis, a long-standing inflammation of the gallbladder, often leads to fibrotic changes and calcification of the gallbladder wall, culminating in the development of a PGB (4). Importantly, this condition can result from various underlying etiologies, including biliary hyperplasia, mucin hypersecretion, and obstruction (4–6, 14). Such pathological changes in the gallbladder may also be associated with other systemic conditions, including pancreatitis, hyperlipidemia, and duodenitis, which can complicate the clinical presentation and management of affected dogs.

PGB is an extremely rare condition in dogs and has been primarily studied in human medicine. To the authors' knowledge, only two cases of PGB have been reported in dogs (15, 16). One was associated with biliary adenocarcinoma at the gallbladder neck, cholelithiasis, biliary obstruction, and lymphoplasmacytic cholecystitis (15), while the other showed no evidence of neoplasia, cholelithiasis, or hepatobiliary inflammation (16). In the present case, although no neoplastic changes were identified, the dog presented with concurrent cholelithiasis and a severe gallbladder mucocele without significant inflammation. Unlike previous reports, this case suggests that chronic gallbladder wall injury due to bile stasis and mucocele formation may contribute to the development or progression of PGB. It also indicates that PGB can occur in the absence of biliary neoplasia or active inflammation. PGB is extremely rare in dogs, and this case study presents an unusual report of a Shih Tzu diagnosed with PGB.

## 2 Case description

A 13-year-old spayed female Shih Tzu presented with anorexia accompanied by vomiting, progressive weight loss, and lethargy. On physical examination, mild tenderness was noted upon palpation of the upper abdomen, with no other significant abnormalities observed.

Laboratory analysis revealed elevations in hepatic enzymes, including a slight increase in alanine aminotransferase (ALT) levels, as well as marked elevations in alkaline phosphatase (ALKP) and gamma-glutamyl transferase (GGT). Hyperlipidemia with concurrent hypercholesterolemia was noted. Additionally, elevated levels of canine pancreatic lipase (cPL) were identified. Total leukocyte count and C-reactive protein (CRP) levels were within reference ranges, as were total bilirubin and serum calcium concentrations (Table 1). Moreover, endocrine testing, including a low-dose dexamethasone suppression test and a thyroid panel (tT4, fT4, and canine thyroid-stimulating hormone [cTSH]) test, was performed to evaluate potential underlying hyperadrenocorticism or hypothyroidism. The only abnormal finding was an elevated cTSH level (Table 1).

**Table 1:** Summary of blood test results on presentation.

**Parameter**	**Patient value**	**Reference range**	**Unit**
Alanine aminotransferase (ALT)	211 ↑	10–125	U/L
Alkaline phosphatase (ALKP)	1083 ↑	23–212	U/L
Gamma-glutamyl transferase (GGT)	149 ↑	0–11	U/L
Aspartate aminotransferase (AST)	49	0–50	U/L
Total bilirubin	0.3	0–0.9	mg/dL
Triglyceride	208 ↑	10–100	mg/dL
Cholesterol	400 ↑	110–320	mg/dL
Canine pancreatic lipase (cPL)	836 ↑	0–200	ng/mL
White blood cell count (WBC)	7.27	5.05–16.76	×10^9^/L
C-reactive protein (CRP)	< 10	0–20	mg/L
Total calcium	10.2	7.9–12	mg/dL
Ionized calcium (iCa)-pH7.4	1.29	1.16–1.47	mmol/L
Total T4 (tT4)	1.5	1.1–4.6	μg/dL
Free T4 (fT4)	1.2	0.6–3.7	ng/dL
Canine thyroid stimulating hormone (cTSH)	1.37 ↑	0.05–0.42	ng/mL
**Cortisol** ^*^			
0 h	4.8	1–6	μg/dL
Post 4 h	0.8	< 1.0	μg/dL
Post 8 h	< 0.5	< 1.0	μg/dL

^*^Method: low-dose dexamethasone suppression test (LDDST).

↑: Increased compared to reference range.

## 3 Diagnostic assessment, therapeutic intervention, follow-up, and outcomes

**Figure 1:**
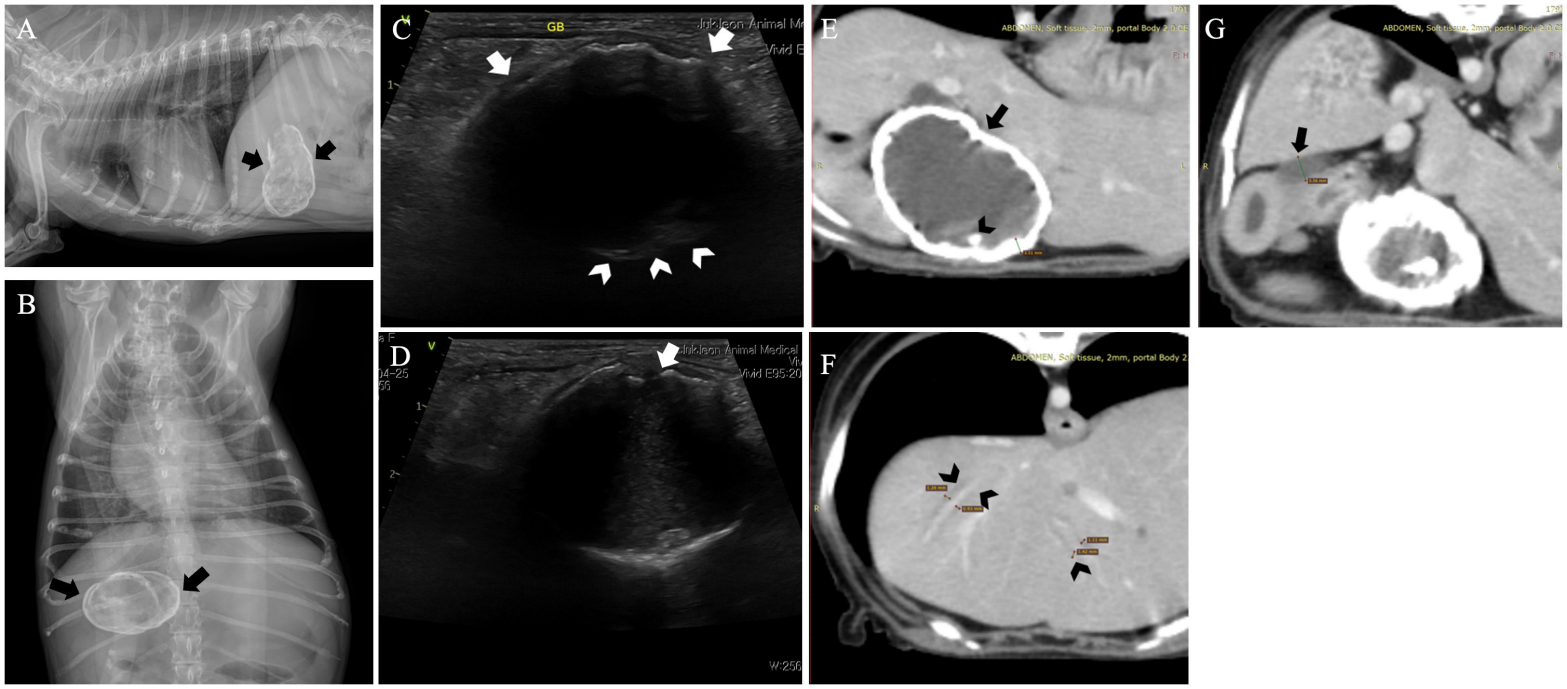
Radiograph images of this dog revealed a nearly spherical calcified structure with irregular margins, suggestive of a calcified gallbladder (arrows). The gallbladder shows both linear and floccular calcifications. **(A)** Right lateral view. **(B)** Ventrodorsal view. Abdominal ultrasound confirmed the presence of a calcified gallbladder wall characterized by posterior acoustic shadowing in this dog. **(C)** A biconvex curved echo structure with variable acoustic shadows shows a curvilinear hyperechoic area along the anterior wall (arrow). In contrast, the posterior wall (arrowhead) is mostly obscured by the posterior acoustic shadowing, with only a part being visible. **(D)** Areas of non-shadowing calcium (arrow) and part of the gallbladder interior are observed. Computed tomography of the dog. **(E)** An increase in the gallbladder wall thickness (arrow) and extensive wall calcification were observed. Cholelithiasis (arrowhead) was noted within the gallbladder. **(F, G)** Mild dilation of the common bile duct (arrow), along with dilation of the intrahepatic bile ducts (arrowhead), was observed.

Abdominal radiographs revealed clear calcification of the gallbladder wall, identified as a nearly spherical structure with linear and floccular mineralization located in the upper right quadrant (Figures 1A, B). Abdominal ultrasonography revealed the gallbladder wall as an irregular, hyperechoic mass with posterior acoustic shadowing, obscuring the gallbladder lumen (Figures 1C, D). Computed tomography (CT) imaging confirmed increased gallbladder wall thickness (4.14 mm) with extensive calcification and the presence of gallstones (Figure 1E). Mild dilation of the common bile duct (5.56 mm) and intrahepatic bile ducts was observed (Figures 1F, G). Pancreatic changes included altered parenchymal volume, cystic lesions, and mild pancreatic duct dilation (1.44 mm), suggestive of pancreatitis, and duodenitis indicated by increased duodenal wall thickness and luminal dilation due to retained fluid. No significant hepatic lymph node abnormalities were observed.

**Figure 2:**
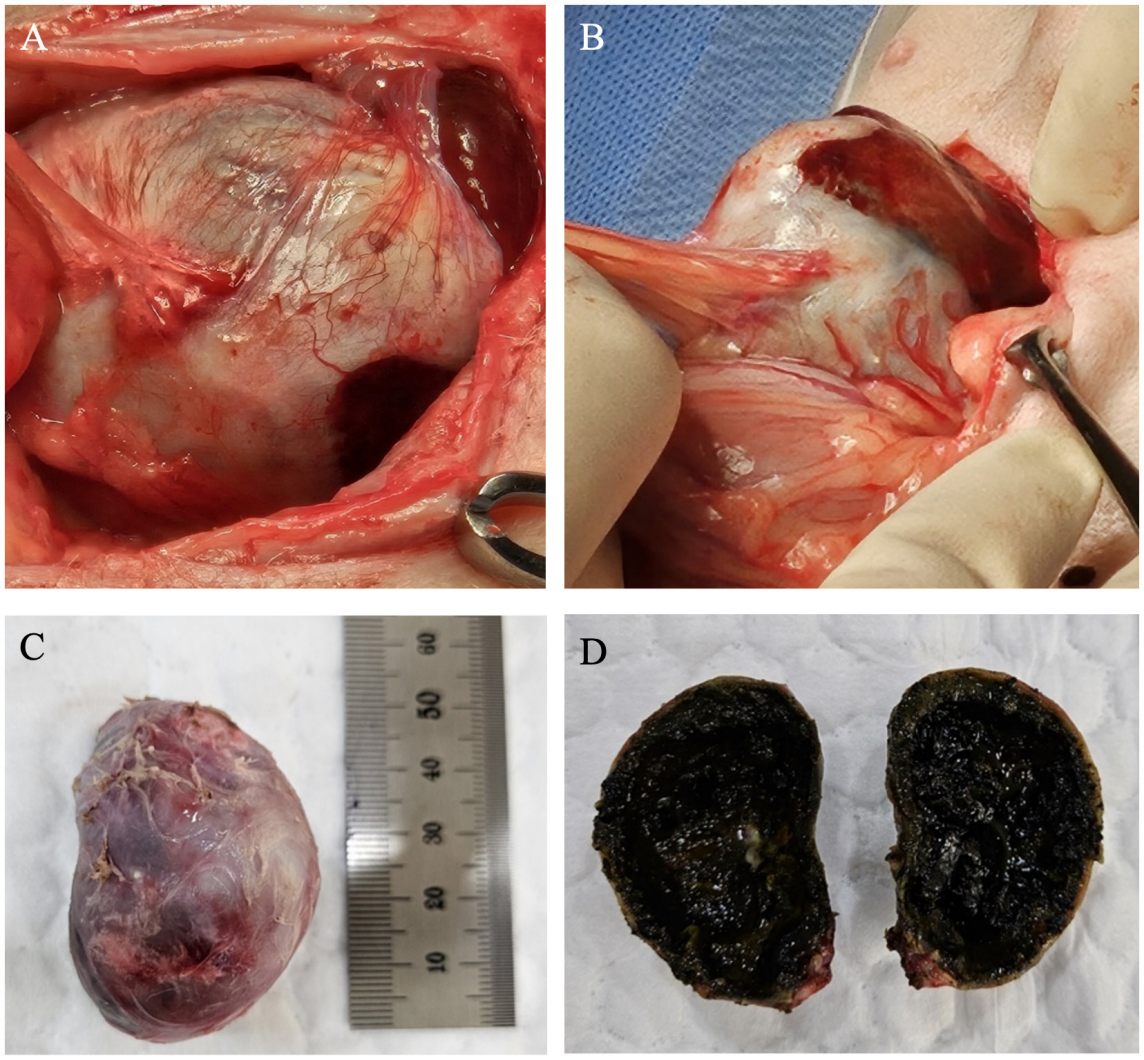
Gross specimen of the gallbladder during and after surgery. **(A, B)** Aspect of calcified gallbladder at operation. The surrounding omentum was severely adhered to the gallbladder, and the gallbladder was firmly attached to the liver. **(C)** The surface of the calcified gallbladder was generally hard, rough, and irregular. Its color ranged from bluish-purple to grayish-white, with certain areas appearing red due to inflammatory changes. **(D)** The interior of the gallbladder exhibited mucoid degeneration and was filled with a dark greenish-black, degenerated jelly-like substance.

A cholecystectomy was performed. Intraoperatively, the gallbladder was found embedded within the liver, with the wall appearing firm and rigid due to extensive calcification, which was diffused throughout the wall, rendering it brittle and difficult to manipulate during surgery. The gallbladder was severely adhered to the surrounding omentum and liver, likely due to prolonged inflammation (Figures 2A, B). Adequate traction of the densely indurated gallbladder wall was difficult, making surgical exposure challenging. The gallbladder was carefully elevated and dissected from the gallbladder neck, while separating the surrounding adhesions. A portion of the hepatic tissue was resected together with the gallbladder due to severe adhesion to the gallbladder wall, which made separation unfeasible. Liver biopsy was also performed for histopathological examination. A gross examination of the resected gallbladder showed slight enlargement (5 × 2.8 cm) with a hard, rough, and irregular surface due to calcification. The overall color was bluish-gray, with areas of red indicating inflammation (Figure 2C). Upon sectioning, the gallbladder wall showed significant thickening and calcification, and the lumen was filled with dark green, gelatinous material indicative of a mucocele (Figure 2D).

**Figure 3:**
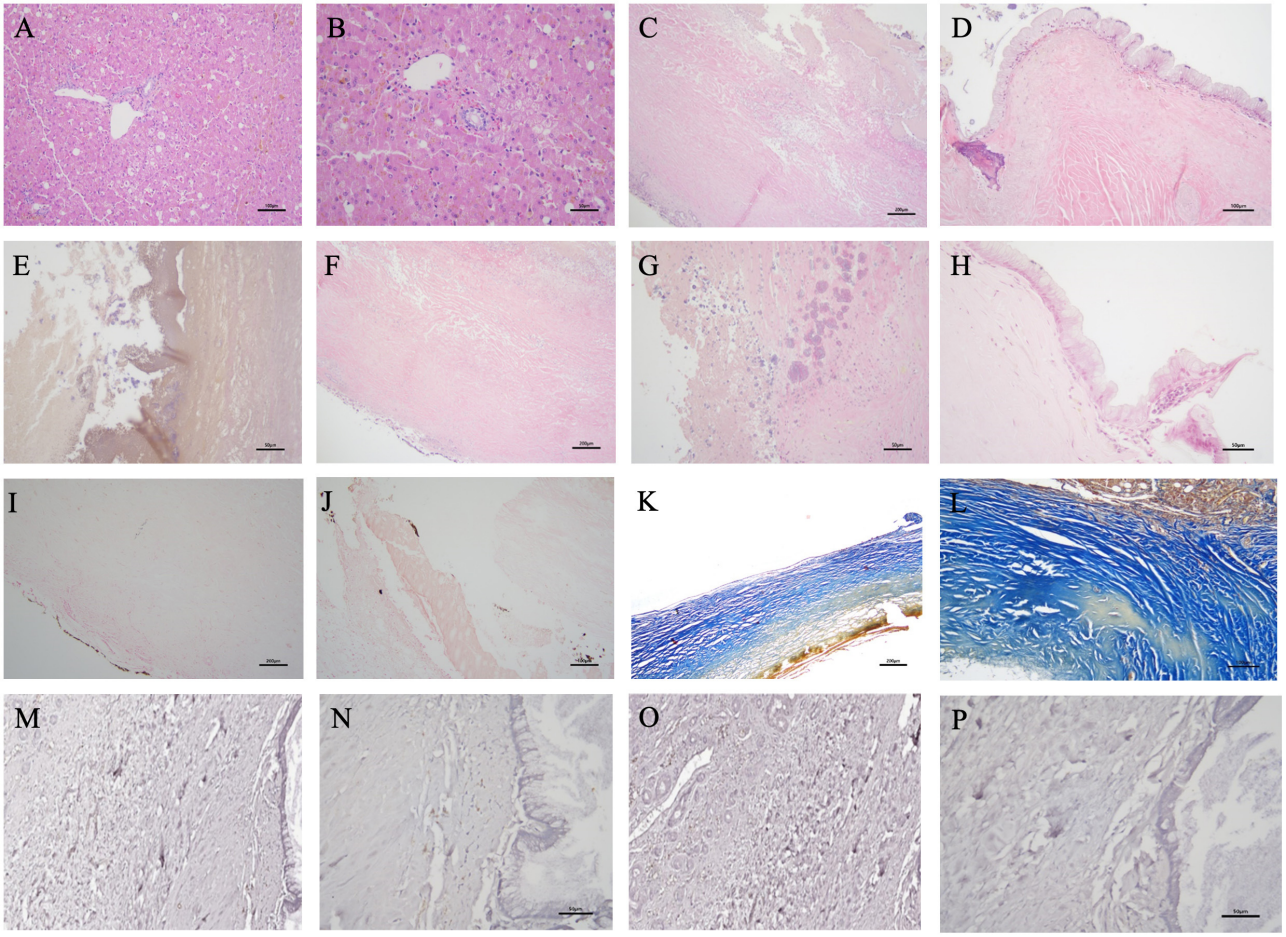
Histopathology of this case obtained from hepatic tissue [Hematoxyl and eosin (H&E) stain, ×200 **(A)**, ×400 **(B)**]. No neoplastic or inflammatory changes originating from hepatocytes or biliary epithelial cells were observed. Vacuolar changes in hepatocytes were distributed multifocally. Mild cholestasis was partially observed. The histopathology of this case obtained from the lesion of the gallbladder wall [H&E stain, ×100 **(C, F)**, ×200 **(D)**, ×400 **(E, G, H)**, Von Kossa stain, ×100 **(I)**, ×200 **(J)**, Masson's trichrome stain, ×100 **(K)**, ×200 **(L)**, Immunohistochemistry stain; Paired Box 5 (PAX5), ×200 **(M)**, ×400 **(N)**, Cluster of Differentiation 3 (CD3), ×200 **(O)**, ×400 **(P)**]. **(C)** Most of the mucosal epithelium was difficult to observe clearly due to ulcerative changes and necrosis, resulting in exfoliation. The entire thickness of the gallbladder wall exhibited severe degeneration, making it impossible to distinguish individual layers. **(D)** Some remaining mucosal epithelium exhibited mild diffuse papillary or irregular proliferation into the lumen, with many epithelial cells in the crypts undergoing mucinous hyperplasia. **(E)** The lumen of the gallbladder contained yellowish or dark greenish-black amorphous materials. Small basophilic amorphous materials, presumed to be minerals or calcium mixed with mucus, are observed on the mucosal surface. **(D, F, G)** Localized areas of ossification or mineral deposits were identified below the mucosal surface. **(H)** Few plasma cells and lymphocytes are observed in the superficial layer of the remaining mucosal propria. **(I, J)** Granular multifocal black- mineralization foci were primarily observed on the gallbladder mucosal surface. **(K, L)** The dense collagen matrix constitutes the majority of the gallbladder wall. The fibrotic connective tissue stained blue, and extensive fibrosis and calcification of the gallbladder wall were observed. Areas of the wall that did not exhibit the blue staining well may indicate calcium deposits or necrotic lesions of the fibrous tissue. The expression of immunohistochemistry stain was very limited, making an inflammatory response unlikely. **(M)** PAX5 positive B lymphocytes were not clearly seen, but **(N)** a few B cells were observed in the preserved mucosal areas. **(O)** CD3 positive T lymphocytes were rarely identified, and **(P)** no T cells were observed in the preserved mucosal areas.

Furthermore, histopathology of the liver revealed no neoplastic or significant inflammatory changes. Multifocal vacuolar changes in hepatocytes were observed, likely due to excessive glycogen deposition (Figures 3A, B). Histopathological examination of the gallbladder wall showed severe changes, with extensive necrosis and ulceration (Figure 3C). The preserved mucosal epithelium exhibited severe papillary hyperplasia and increased mucus secretion (Figure 3D), with the gallbladder lumen containing yellowish or dark greenish-black amorphous materials indicative of a gallbladder mucocele (Figure 3E). Most of the mucosal epithelium was ulcerated or necrotic, hindering detailed observation. The entire gallbladder wall exhibited severe alterations, obliterating the distinction between lamina propria, muscularis propria, and perimuscular connective tissue. Additionally, the muscular layer lacked normal fiber bundle organization (Figure 3C), and the serosal layer included adherent liver tissue. Small basophilic amorphous materials were identified on the mucosal surface, with suspected ossification or mineral deposition beneath (Figures 3D–G). Von Kossa staining revealed multifocal mineralization foci on the mucosal surface of the gallbladder with multiple ulcers (Figures 3I, J), confirming a diagnosis of PGB. Masson's Trichrome staining showed a dense collagen matrix replacing most of the gallbladder wall with no clear muscle fiber bundles. The fibrous connective tissue stained blue, and regions that did not stain well indicated calcium deposition. Notably, extensive fibrosis was evident throughout the gallbladder wall (Figures 3K, L). Hematoxylin and eosin staining showed few plasma cells and inflammatory cells in the remaining lamina propria (Figure 3H). Immunohistochemical analysis showed weak CD3 (cluster of differentiation 3) staining, indicating few T lymphocytes, and PAX5 (paired box 5) staining revealed few B lymphocytes in preserved mucosal areas, not suggestive of a significant inflammatory response (Figures 3M–P). Transmural necrosis was widespread, and the lesion appeared chronic, with no active cholecystitis observed. Aerobic, anaerobic, and fungal cultures of the bile revealed no significant pathogens.

Combining imaging findings, gross evaluation, and histopathological results, PGB due to chronic wall damage, necrosis, ulceration, fibrosis, and calcification, along with a severe mucocele, was diagnosed.

Postoperatively, the patient received symptomatic treatment with Lactated Ringer's solution for fluid therapy. For liver protection and function improvement, the patient was treated with ursodeoxycholic acid at 7.5 mg/kg bid, silymarin at 10 mg/kg bid, and S-adenosyl methionine at 100 mg/head sid. Gastrointestinal symptoms were managed with antiemetic and gastric acid suppression therapy using maropitant at 1 mg/kg sid and omeprazole at 0.5 mg/kg bid. Furthermore, ALKP and GGT levels gradually decreased, while ALT and AST levels initially raised post-surgery but then decreased to normal ranges. CRP levels increased post-surgery but quickly normalized, likely due to tissue damage and inflammatory response from the surgery. Mild dilation of the common bile duct was noted on follow-up ultrasound, which is considered an adaptive change. The patient recovered without postoperative complications and remained well-recovered for more than 2 months without clinical symptoms or elevated liver enzyme levels after symptomatic treatment discontinuation.

## 4 Discussion

This case study discusses a rare instance of PGB in a dog, marked by severe calcification of the gallbladder wall, chronic fibrosis, and extensive gallbladder mucocele. Diagnostic imaging and histopathology were essential for diagnosis and comprehension of the condition. Cholecystectomy resolved symptoms and normalized liver enzymes. Although gallbladder cancer (GBC) incidence in PGB cases is low, the evident link to gallbladder cancer warrants consideration of prophylactic cholecystectomy.

The term PGB is used to emphasize the bluish discoloration and brittle consistency of the gallbladder wall observed during surgery (1, 5, 7, 17). Its diagnosis is primarily achieved through imaging studies, which reveal the characteristic calcification and thickening of the gallbladder wall (2, 14, 18, 19). Additional diagnostic evaluations, including blood tests and histopathological examinations, are essential to assess the extent of hepatic and pancreatic involvement and to rule out neoplastic changes (7, 18).

In veterinary medicine, PGB is extremely rare. The first reported case of PGB in dogs involved a 9-year-old spayed female Poodle with chronic proliferative lymphoplasmacytic cholecystitis, calcification, and a well-differentiated adenocarcinoma at the gallbladder neck (15). The second reported case occurred in an 11-year-old neutered male Border terrier, in which no evidence of neoplasia, cholelithiasis, or hepatobiliary inflammation was observed. The dog was diagnosed with diabetes mellitus with ketoacidosis, pancreatitis, and peritonitis. The clinical condition of the patient deteriorated rapidly within 24 h of diagnosis, and euthanasia was elected by the owner. Based on imaging findings and postmortem examination, the PGB was considered an incidental finding (16). This report presents the first case report describing the pathological features of PGB associated with gallbladder mucocele and chronic fibrous changes in the gallbladder wall in a Shih Tzu dog, regardless of tumor presence. In human medicine, it is a rare condition, occurring in 0.06% to 0.8% of cholecystectomy specimens (12, 20). Moreover, over 95% of reported cases exhibited PGB associated with gallstones (21). Traditionally, the association between PGB and GBC has been well recognized in humans, with calcification of the gallbladder wall acting as a risk factor for GBC. The incidence of GBC in PGB has been reported to range between 12% (7) and 33% (18). However, more recent studies have reported a lower incidence between 0% (22) and 5% (23). Therefore, the relationship between GBC and PGB remains unclear. Notably, studies on the association between PGB and cancer in dogs are lacking, and no evidence of cancer was found in this case. Nevertheless, the presence of PGB indicates the need for cholecystectomy, which can help prevent potential complications. In humans, PGB is known to occur through various mechanisms, primarily related to chronic inflammation causing hemorrhage, ischemia, scarring, and hyalinization of the wall, and can be induced by factors such as gallstones, cystic duct obstruction, and calcium metabolism imbalance (3, 5, 7, 8, 13, 17, 24). This condition is generally considered a form of dystrophic calcification, characterized by the deposition of calcium and other mineral salts in necrotic or non-functional tissues, even in the presence of normal serum total calcium and ionized calcium levels (15). In both the previously reported canine case and the present case, hypercalcemia was not observed (15, 16). In the present case, cholelithiasis, severe gallbladder mucocele, and bile stasis were identified. These changes may have contributed to gallbladder wall injury and impairment of its absorptive and secretory functions, potentially leading to mineral deposition and calcium salt saturation within the bile. Saturation of bile by calcium salts is essential for the subsequent formation of calcium precipitates (25). Although the exact etiology of PGB remains unclear in previously reported cases, similar mechanisms involving dystrophic calcification of structurally compromised gallbladder walls may underlie the development of PGB in dogs.

Based on the extent, gallbladder calcification is classified as complete intramural or selective mucosal calcification (23, 26). In the complete type, the gallbladder wall undergoes total calcification and fibrosis, while in the selective type, calcification is milder and confined to the mucosal layer. In a study of 44 PGB patients, 17 and 27 exhibited complete intramural and selective mucosal calcification, respectively. None of the patients with the complete type had GBC, whereas two individuals with the selective type had GBC, suggesting a possible association between localized calcification and PGB with GBC (23). Furthermore, histopathological examination of current case revealed severe papillary hyperplasia of the gallbladder mucosal epithelium and excessive mucus secretion, leading to gallbladder mucocele diagnosis. Additionally, multiple ulcers were identified on the mucosal and serosal surfaces, accompanied by calcification along the mucosal surface, diffuse fibrosis, and some calcific changes in the gallbladder wall. This reflects chronic pathological changes in the gallbladder, including cholecystitis, indicating tissue damage and regeneration processes that can impair gallbladder function (27). Among 219 gallbladders with chronic cholecystitis in dogs, mucosal lymphoplasmacytic infiltration was observed in 189 cases (86.3%). However, lymphocytes and/or plasma cells were absent in 18 cases (18/219; 8.2%). In 12 dogs, necrosis or ulceration prevented detailed mucosal examination (27). Here, of the mucosal epithelium was severely altered by ulceration and necrosis, obscuring the structure and making it difficult to distinguish the gallbladder wall's different layers. In the preserved mucosal areas, few inflammatory cells were observed. While no active cholecystitis was evident, the chronic nature of the lesions implies prior histological changes from chronic cholecystitis.

PGB diagnosis is primarily made through imaging studies and can often be an incidental finding during abdominal imaging. Simple abdominal radiographs may show a rim-like calcification of the gallbladder wall (13, 20, 28). The radiographic appearance of a calcified gallbladder varies depending on the location, extent, and degree of calcification. Less severe calcification may be difficult to identify on plain radiographs, whereas more extensive calcification appears as rounded or curvilinear patterns (28). Ultrasound and CT scans can evaluate the location and extent of gallbladder wall calcification (18, 29–31). In PGB, ultrasound findings are classified into three types based on calcification characteristics. Type I, the sclerotic atrophic pattern, features a highly echogenic crescent with a complete posterior acoustic shadow. Type II displays a biconvex curved echo structure, with variable acoustic shadows that are less intense than Type I, permitting visibility of the anterior and posterior walls either fully or partially. Type III presents as an irregular echogenic mass with a posterior acoustic shadow that is similar yet less pronounced than Type I. Type I indicate complete intramural calcification, while Types II and III suggest variations of selective mucosal calcification (18). In our case, the patient primarily exhibited variations of selective mucosal calcification corresponding to Types II and III. As CT is the most sensitive method for detecting calcification, PGB is often over diagnosed on CT. Therefore, clinicians must be aware of common pitfalls in imaging evaluation and perform careful assessments (32). Here, gallbladder wall calcification was initially detected on abdominal radiographs, confirmed, and further evaluated using ultrasound and CT scans. Gallbladder wall thickening, calcification, and the presence of gallstones were observed, which are typical findings of PGB.

Further, this case demonstrates that gallbladder mucocele and PGB can occur simultaneously. Gallbladder mucocele is characterized by hyperplasia of the mucin-secreting glands in the gallbladder mucosa and abnormal accumulation of mucus within the gallbladder lumen (33–36). When bile-laden mucus spreads into the cystic, hepatic, and common bile ducts, it may cause different levels of extrahepatic biliary obstruction. The gallbladder can distend from mucocele, causing pressure necrosis of its wall and risking rupture (37). Finally, obstruction in the cystic duct from mucocele can also lead to calcium salt deposits in the mucosa, possibly leading to PGB development.

PGB treatment usually involves surgical resection to prevent complications from calcification and chronic changes in the gallbladder wall. PGB is an extremely rare condition in dogs, which limits the ability to accurately assess the prognosis following cholecystectomy. Because PGB is characterized by irreversible pathological changes, delayed surgical intervention may result in serious complications such as gallbladder rupture, bile peritonitis, and sepsis. In the absence of malignancy, the surgical outcome may be comparable to that of cholecystectomy performed for other biliary disorders. However, comorbidities such as septic peritonitis, pancreatitis, hepatic failure, or renal failure may adversely affect surgical prognosis (38, 39). Although therapeutic alternatives are limited, conservative or medical management may be appropriate in asymptomatic patients or those with high surgical risk. Impaired bile flow has been shown to increase the risk of bacterial translocation across the intestinal mucosa. Therefore, in cases where hepatobiliary infection is suspected, the administration of broad-spectrum antimicrobial therapy should be considered (40). Adjunctive therapies, including ursodeoxycholic acid, S-adenosylmethionine (SAMe), and antioxidants, may promote bile flow, reduce bile viscosity, and offer anti-inflammatory and antioxidant benefits (40–43). Given that high-fat diets may aggravate gallbladder disease, a low-fat, highly digestible diet is recommended (40). Furthermore, appropriate symptomatic management, including antiemetics and analgesics, should be implemented as needed.

This study highlights the rare association between PGB and gallbladder mucocele in dogs, with observed improvements in clinical symptoms and liver enzyme levels after cholecystectomy. These findings emphasize the importance of timely diagnosis and surgical intervention. Further studies are needed to elucidate the pathological mechanisms underlying PGB, gallbladder mucocele, and their potential interrelationship. Understanding these mechanisms may support earlier disease prediction and improve diagnostic and therapeutic approaches.

## Data Availability

The original contributions presented in the study are included in the article/supplementary material, further inquiries can be directed to the corresponding author.
